# Taxonomic and functional profiles of soil samples from Atlantic forest and Caatinga biomes in northeastern Brazil

**DOI:** 10.1002/mbo3.169

**Published:** 2014-04-04

**Authors:** Ralfo G Pacchioni, Fabíola M Carvalho, Claudia E Thompson, André L F Faustino, Fernanda Nicolini, Tatiana S Pereira, Rita C B Silva, Mauricio E Cantão, Alexandra Gerber, Ana T R Vasconcelos, Lucymara F Agnez-Lima

**Affiliations:** 1 Department of Cellular Biology and Genetics, UFRNNatal, Rio Grande do Norte, Brazil; 2 National Laboratory of Scientific ComputingQuitandinha, Petrópolis, RJ, Brazil; 3 Centro de Biotecnologia, Universidade Federal do Rio Grande do SulPorto Alegre, RS, Brazil; 4 Empresa Brasileira de Pesquisa AgropecuáriaEmbrapa Suínos e Aves, Concórdia, Santa Catarina, Brazil

**Keywords:** Atlantic forest, bioinformatic, caatinga, comparative metagenomics, pyrosequencing.

## Abstract

Although microorganisms play crucial roles in ecosystems, metagenomic analyses of soil samples are quite scarce, especially in the Southern Hemisphere. In this work, the microbial diversity of soil samples from an Atlantic Forest and Caatinga was analyzed using a metagenomic approach. Proteobacteria and Actinobacteria were the dominant phyla in both samples. Among which, a significant proportion of stress-resistant bacteria associated to organic matter degradation was found. Sequences related to metabolism of amino acids, nitrogen, and DNA and stress resistance were more frequent in Caatinga soil, while the forest sample showed the highest occurrence of hits annotated in phosphorous metabolism, defense mechanisms, and aromatic compound degradation subsystems. The principal component analysis (PCA) showed that our samples are close to the desert metagenomes in relation to taxonomy, but are more similar to rhizosphere microbiota in relation to the functional profiles. The data indicate that soil characteristics affect the taxonomic and functional distribution; these characteristics include low nutrient content, high drainage (both are sandy soils), vegetation, and exposure to stress. In both samples, a rapid turnover of organic matter with low greenhouse gas emission was suggested by the functional profiles obtained, reinforcing the importance of preserving natural areas.

## Introduction

Despite soils being the largest reserve of microbial biodiversity, our knowledge about their genetic pool is still limited (Whitman et al. 1998; Torsvik et al. 2002; Curtis and Sloan 2004; Mocali and Benedetti 2010). In general, soil microbial diversity has been mainly accessed through methods based on 16S gene analysis that allow for estimating the diversity richness, but fail in the identification of functional attributes. Thus, metagenomic studies have contributed toward the understanding of microbial diversity allowing for the identification of taxa and functional profiles, as the abundance of determinate genes have been used as an indicator of biogeochemical processes (Morales et al. 2010; Brankatschk et al. 2013).

Soil characteristics are important factors that affect the microbial diversity and the dynamics of biogeochemical cycles. As an example, soil pH and nutrient availability have been considered the main factors driving the composition of soil bacterial communities (Lauber et al. 2009; Goldfarb et al. 2011; Griffiths et al. 2011; Kuramae et al. 2012). In addition, high temperature, moisture, low pH, and carbon and nitrogen availability contribute to the increase in N_2_O emission from soil, but aeration and drought cause reduction in this process (Sangeetha et al. 2009; Cleveland et al. 2010; Saggar et al. 2013). Even so, our knowledge about biogeochemical processes is still scarce, mainly in relation to tropical soils, and questions about how the soil characteristics together determine the microbial diversity and biogeochemical processes remains unclear.

With climate changes and the increase in greenhouse gas emission, the understanding of microbial diversity and its involvement in these processes will be important for adequate soil management. The highest emissions of CH_4_ and N_2_O are from tropical soils, but these soils also present high consumption of CO_2_ (Montzka et al. 2011). However, the majority of these data are from humid tropical regions and little is known about semiarid tropical soils.

In this work, the microbial diversity of soil samples from Parque das Dunas (PD), as a representative of Atlantic Forest biome, and João Camara city, as representative of Caatinga biome, both located in the State of Rio Grande do Norte (RN, Brazil), were investigated using a metagenomic approach. The Atlantic forest is the third largest Brazilian biome, comprising particular ecosystems such as mangroves and forests which span almost the entire Brazilian coast, and is the second largest humid tropical forest in South America. However, less than 10% of the native forest is preserved (Myers et al. 2000) and few works have investigated their microbial diversity. The analysis of 16S gene in soil samples from Atlantic Forest of Paraná and Rio de Janeiro showed a dominancy of Acidobacteria and Proteobacteria (Bruce et al. 2010; Faoro et al. 2010). Concerning to gas emission, the analysis of soil samples of Atlantic Forest from São Paulo showed that N_2_O emission and the CH_4_ uptake are within the range of other tropical forests of the world. The authors also observed that N_2_O and CO_2_ emissions were lower at higher altitudes, which may be associated to the temperature decrease (Sousa Neto et al. 2011). The PD in Natal city (northeastern Brazil) differs from other representatives of Atlantic Forest due to its soil characteristics. The forest growth on the dunes suggests the occurrence of a specific microbial diversity, differing of the microbiota observed in soil from Atlantic forest from Paraná and Rio de Janeiro, which are more clayey soils.

João Câmara (JC) sampling site is located in the semiarid Caatinga, the only exclusive Brazilian biome. The Caatinga occupies 18% of the Brazilian territory, being the most populous semiarid region of the world. It presents a rich diversity of plants, but only 47% of the native vegetation is preserved. Due to natural and anthropogenic factors, the Caatinga is considered a fragile ecosystem subject to desertification. Despite the biological importance, the microbial diversity of Caatinga soils is still unknown, but due to severe climate conditions (high temperature, high UV exposure, and long periods of drought), a low and specialized microbial diversity was estimated (Giongo et al. 2011; Menezes et al. 2012).

Although the Atlantic Forest and Caatinga in northeastern Brazil have specific soil characteristics (both are sandy soils poor in nutrients) and an endemic flora, we investigated the hypothesis that soil microbes show a distinct taxonomic and functional composition in relation to other biomes.

Reports of microbial diversity are scarce in the Southern Hemisphere, providing an invaluable, interesting, and unexplored field of study for metagenomics. Therefore, to our knowledge, this is the first metagenomic study ever conducted in soils of Caatinga and Atlantic Forest biomes that describe taxonomic and metabolic profiles of the microbial community, as well as a comparative analysis between these two different environments and other public metagenomes from different biomes.

## Materials and Methods

### Sample collection

Sampling was performed in early October 2009 in two different regions of Rio Grande do Norte state, Brazil: the Parque das Dunas (Park of Dunes, PD) in Natal city, an environmental conservation area of Atlantic Forest biome (1172 ha) covered by native coastal dune vegetation, and João Câmara city (JC), a semiarid area of Caatinga biome 73 km from Natal (Table 1 and Figure S1). The collection site at PD (S5° 50.530′ O35° 11.598′) is located on uneven ground, is grayish in color, under indirect sunlight, and has many roots. The collection site in JC (S5° 30′ 51.81″ O35° 54′ 17.13″) is located on flat ground, is dry, dark brown, under direct sunlight, and has few roots (Table 1). The sampling followed Schneegurt et al. (2003) recommendations. In brief, after the removal of roots, ∼500 g of soil samples were collected (depth 5–10 cm) using a sterile spatula and immediately transferred to sterile 50 mL tubes kept on ice. Organic and physical–chemical analyses of the soil samples were performed by the Laboratory of Soil, Water and Plants Analyses of EMPARN (Natal, RN, Brazil).

**Table 1:** General features of the studied areas

	Parque das Dunas (PD)	João Câmara (JC)
Biome	Atlantic forest	Caatinga
Climate zone	Humid tropical	Semiarid
Temperature (annual average)	22.6–29.2°C	21–32°C
UV	High, but indirect due to canopy	High and direct
Rainfall (annual average)	1600 mm	648 mm
Vegetation	Predominantly arboreal, also presenting shrubs and herbaceous. The main families found are Leguminosae, Myrtaceae, Gramineae (Poaceae), Compositae, Euphorbiaceae, Convolvulaceae, and Rubiaceae (Freire 1990)	Xerophilous species, shrubs, thorny and deciduous small trees (Santos et al. 2010)
Soil	Low compaction, sandy, grayish	Compact, dry, dark brown, with few roots

### DNA preparation, extraction, purification, and sequencing

Initially, samples were sieved through 2 mm sterile sieves in order to eliminate undesired constituents such as roots and tiny stones. Afterwards, 10 g of soil samples were subjected to direct extraction and purification of DNA using Power MaxTM Soil DNA Isolation Kit (MoBio Laboratories, Inc., Carlsbad, CA) following the manufacturer's instructions.

For sequencing, the libraries were prepared following the instructions of the GS FLX titanium general library preparation method manual (454-Roche), using 5 *μ*g DNA of each sample. The titration, emulsion PCR, and sequencing steps were performed according to the manufacturer's instructions. A four-region 454 sequencing run was performed on a 70 × 75 PicoTiterPlate (PTP) using the Genome Sequencer FLX System (Roche Applied Science, Sao Paulo, Brasil) (Margulies et al. 2005). Each library was loaded onto one quarter of the plate. The sequences were deposited in GenBank with accession numbers SRA026684 and SRA026685 and on the MG-RAST server (4459906.3 and 4459907.3).

The replicated sequences generated as artifact of the 454-based pyrosequencing, were eliminated using the Replicates software (Gomez-Alvarez et al. 2009), and the sequences <120 bp were removed by the LUCY program (Chou and Holmes 2001). As a result, 27,618 and 22,611 reads were removed in PD and JC, respectively.

The assembly was conducted using the Newbler Assembler 2.5.3. Reads identified as Partial, Repeat, Outlier, TooShort, and with high-quality discrepancies were filtered from the dataset. Assembling and filtering cycles were performed until the discrepancies were limited to 1% of the total of the reads filtered out at the first assembly step. However, at the cutoff threshold no contigs were assembled.

### Taxonomic distribution and statistical analyses

The taxonomic profiles of the metagenomic reads were assigned using the MG-RAST server. In MG-RAST, the species richness was computed as the antilog of the Shannon diversity (Meyer et al. 2008). The abundance data was identified through the lowest common ancestor (LCA), with the parameters 1e^−05^ as the maximum e-value, a minimum identity of 60%, and a minimum alignment length of 15 as cutoff. The statistical analysis for distinct taxonomic levels from MG-RAST was conducted using the Statistical Analyses of Metagenomic Profiles (STAMP) (Parks and Beiko 2010) software. The significance of the relative proportion difference in taxonomic distribution of PD and JC samples was performed using the two-sided Fisher′s exact test, with Newcombe–Wilson confidence interval method. Because *P*-values were not uniformly distributed using Storey′s false discovery rate (FDR), Benjamin–Hochberg FDR was applied for correction. Results with *q* < 0.05 were considered significant and the unclassified reads were removed from analyses. The biological relevance of the statistic taxa was determined applying a difference between the proportions of at least 1% and a twofold ratio between the proportions.

A taxonomic analysis was also conducted using the MEGAN4 (Huson et al. 2011) software. The given reads were compared against the NR and NT NCBI databases using the BLASTX and BLASTN algorithms (Altschul et al. 1997), respectively. Statistical tests on the taxonomic data were also performed with MEGAN. The PD and JC counts were normalized to produce data sets of 100,000 reads. The analysis was performed comparing distinct hierarchical levels and directed homogeneity test was applied to highlight the significant differences in the sample comparisons. The highlighting thickness is logarithmically proportional to its significance; that is the thickness is an integer value of 2log *x* when *P* = 1.0ex (Mitra et al. 2009). Multiple testing correction analysis was not applied and all unassigned reads were ignored.

### Functional analyses

Functional profiles were identified using the SEED subsystems annotation source of the MG-RAST, with 1e^−05^ as maximum e-value, a minimum identity of 60%, and a minimum alignment length of 15. Distinct functional levels from MG-RAST were statistically analyzed in the STAMP, using the same parameters above described for taxonomic distribution. Through the workbench tool from MG-RAST server, we generated subsets of the reads annotated in a functional subsystem for taxonomic identification. The metabolic pathways of biogeochemical cycles were generated using the Kyoto Encyclopedia of Genes and Genomes (KEGG) from MG-RAST server, with 1e^−05^ maximum e-value cutoff, 60% of minimal identity, and a minimal alignment length of 15.

A functional analysis using the SEED (Overbeek et al. 2005) and KEGG (Kanehisa et al. 2012) databases was conducted using the MEGAN4 software. Each sequence was related to its SEED functional role using the best BLAST score to protein sequences without known functional roles. A similar procedure was used to match each sequence to a KEGG orthology (KO) accession number.

A database was constructed using proteins' Refseqs for a number of key enzymes of different and important metabolic pathways. A screening of functional key enzymes was conducted by BLAST using the Refseqs against the PD and JC metagenome. Matches with alignment scores higher than 80 were retained.

### Comparative metagenomic analysis

The taxonomic and SEED subsystems profiles of metagenomic samples from soil, water, and host-associated samples were obtained from MG-RAST server. Samples belonging to rhizosphere, temperate and tropical forests, marine habitat, host-associated, and desert biomes were included. The criteria applied for inclusion were a maximum e-value cutoff of 1e-05, a minimum identity of 60%, and a minimum alignment length of 15. The metagenomes included in the analysis were 4440463.3, 4440939.3, 4444130.3, 4444164.3, 4444165.3 (animal-associated habitat), 4465556.3, 4449956.3 (rhizosphere biome), 4477805.3, 4477872.3, 4477901.3, 4477904.3 (desert biome), 4443713.3, 4441057.4, 4441586.3, 4441578.3 (marine habitat), 4477876.3, 4477877.3, 4477899.3 (temperate forest), 4477807.3 and 4477875.3 (tropical forest). Trends in the abundance of the taxonomy and the SEED subsystems were examined using Principal Component Analysis (PCA) through the multiple groups analysis of STAMP, in which the statistical test applied was analysis of variance with Games–Howell post hoc test and Benjamin–Hochberg FDR for correction. For the comparison between two groups, the Welch′s t-test, the Welch′s inverted test for confidence interval method and Benjamin–Hochberg FDR for correction were applied. The relative proportion difference in functional distribution of PD and JC samples was considered significant when *q* < 0.05. The unclassified reads were removed from analyses.

## Results

### Organic and physical–chemical characteristics of soil samples

The organic and physical–chemical parameters of PD and JC soil samples are summarized in Table 2. Both samples are classified as very acidic soils (pH < 5.0) and exhibit a large portion of sand, particularly in PD. However, the PD soil shows lower values for all the components analyzed and higher C:N ratio when compared to the JC sample, an indicator of a deficiency in nutrients and minerals.

**Table 2:** Organic and physical–chemical parameters of PD and JC soil samples

		Ca	Mg	Al	H + Al	P	K	Na	N	Organic matter	
		Ca	Mg	Al	H + Al	P	K	Na	N	Organic matter	
					
Sample	pH in water	(cmol.dm^−3^)				(mg.dm^−3^)			(g.dm^−3^)		C:N
PD	4.99	0.30	0.14	0.10	1.32	2	12	4	0.31	11.26	21:1
JC	4.60	9.5	23.5	2.25	7.76	4	305	199	1.10	24.6	12.7:1

Granulometry (%)	Sand	Clay	Silt	Texture classification
PD	97.9	2	0.1	Sandy
JC	58.2	12	29.8	Sandy loam

### General characteristics of the metagenomes

The soil DNA sequencing resulted in 147,278 and 151,274 reads from PD and JC, with a total of 64,214,298 and 68,328,253 bb, an average length of 436 ± 63 and 451 ± 60 bp, and the GC content of 61 ± 8 and 66 ± 7%, respectively (Table S1). Reads identified as artificial duplicate was removed by the MG-RAST. After the quality control, 155,805 proteins were predicted for PD sample and ∼53% of the total reads were annotated as proteins functionally assigned. For JC metagenome, 164,449 proteins were predicted and 61.6% of the total reads were identified functionally (Table S1). The algorithm implemented by MEGAN assigned more sequences when compared to MG-RAST and it was able to identify a higher number of sequences related to *Bacteria, Archaea, Eukarya*, and *Viruses* (Tables S1 and3), probably due to the higher number of available sequences in the reference database. The species richness estimated through the *α*-diversity index showed that the metagenome from JC presents *α*-diversity of 448.587, whereas for PD the species richness is 441.864.

**Table 3:** Taxonomic profile of PD and JC samples to domain level, computed by MEGAN and MG-RAST

Domain	MEGAN		MG-RAST	
		
	PD	JC	PD	JC
Archaea	266	662	232	366
Bacteria	88,786	106,396	74,299^1^	93,371^1^
Eukaryota	7489	794	5312^1^	701^1^
Viruses	11	25	5^1^	21^1^

1 Differences statistically significant between PD and JC samples (*P* < 1e^−15^) by STAMP.

### Comparative taxonomic profiles

For phylum level, the microbiota profile generated by MG-RAST was similar in the PD and JC samples. The more abundant phylum in both samples were Actinobacteria (27.8% and 36.4%), Proteobacteria (26.1% and 24.8%), and Acidobacteria (9.1% and 2.4%). However, the statistic difference was observed for Proteobacteria, Acidobacteria, and Chlamydia, more frequent in PD sample, and Actinobacteria, Bacteroidetes, and Cyanobacteria with highest frequency in JC. Concerning Archaea and Eukarya phyla, the highest occurrence of Thaumarchaeota was observed in JC sample, while Ascomycota was predominant in PD sample. The MEGAN analysis showed similar results for phylum representation (data not shown).

**Figure 1:**
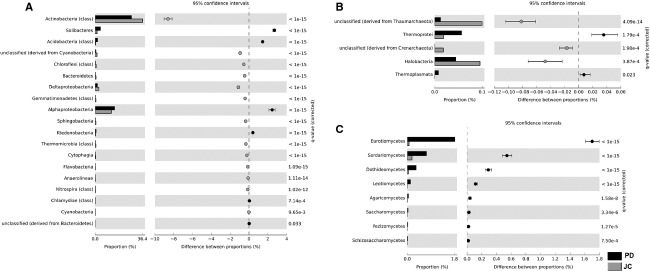
Comparative taxonomic profile of the PD and JC samples at class level, computed by MG-RAST. Classes with significant biological differences (*P* < 0.05, difference between the proportions >1% and twofold of ratio between the proportions, STAMP) for the Bacteria domain (A); for the Archaea domain (B); and for the Eukaryota domain (C).

The Actinobacteria (27.8% and 36.4%) and Alphaproteobacteria (14.86% and 12.41%) were the predominant classes in PD and JC. The classes Alphaproteobacteria, Solibacteres, and Acidobacteria are more frequent in PD sample (Fig. 1), with the highest representation of the orders Rhizobiales, Solibacterales and Acidobacteriales according to STAMP analysis. In JC sample, Actinobacteria, Deltaproteobacteria, and unclassified Cyanobacteria were predominant (Fig. 1), with overrepresentation of the orders Actinomycetales, Sphingomonadales, and Mixococcales. The statistical analyses implemented by MEGAN showed that PD metagenome has a significantly higher number of reads related to Acidobacteria, Alphaproteobacteria, and Planctomycetia classes. Moreover, Betaproteobacteria, Gammaproteobacteria, and Deltaproteobacteria are statistically more represented in the JC metagenome (Figure S2).

**Figure 2:**
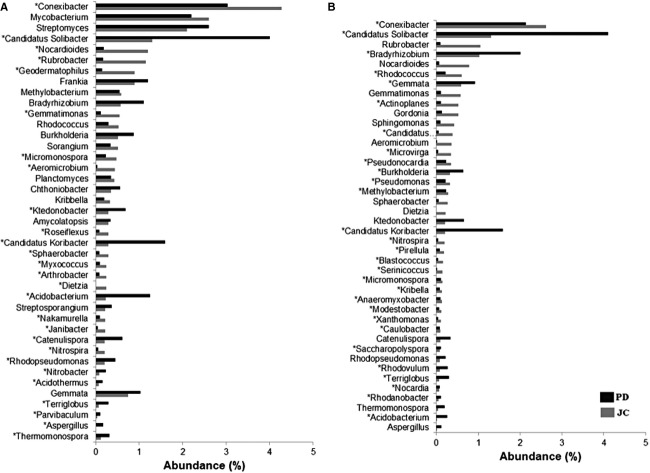
Comparative taxonomic profile of the PD and JC samples at genus level, computed by MG-RAST (A) and MEGAN (B). The twenty most abundant genera found in each samples are shown. *Significant differences between PD and JC samples (*P* < 0.05, difference between the proportions >1% and twofold of ratio between the proportions).

Both metagenomes showed a similar microbial composition considering some of the most frequently found genera, despite the difference in their representation (Fig. 2). The PD sample showed predominance of *Candidatus solibacter, Candidatus koribacter, Acidobacterium* (Acidobacteria)*, Ktedonobacter* (Chloroflexi), and *Catenulispora* (Actinobacteria). In JC sample, the genera with the significant representation were *Conexibacter, Nocardioides, Rubrobacter, Geodermatophilus* (Actinobacteria), and *Gemmatimmonas* (Gemmatimonadetes) (Fig. 2). For PD, *Bradyrhizobium, Burkholderia, Microvirga* (Proteobacteria)*, and Gemmata* (Planctomycetes) were significant only considering the MEGAN analysis. Additionally, *Rhodococcus, Actinoplanes* (Actinobacteria), and *Candidatus choracidobacterium* (Acidobacteria) were indicated as statistically meaningful for JC metagenome by MEGAN (Fig. 2).

Among Archaea classes, Halobacteria (Euryarchaeota phylum) and Thermoprotei (Crenarchaeota phylum) showed significant results in JC and PD metagenomes, respectively, although with low occurrence (Fig. 1).

The PD and JC samples presented a wide variation in hits for Eukaryota domain and low occurrence of viruses. Despite that there was a similar representation of the most abundant phyla in the two metagenomes, the number of sequences assigned to Eukaryota was around 10-fold higher in PD. The major contribution to Eukaryota microbiota in PD came from Ascomycota phylum, with predominance of Eurotiomycetes, followed by Sordariomycetes and Dothideomycetes classes (Fig. 1). Among Eurotiomycetes, *Aspergillus* was the most frequent genus, with 0.171% of all hits for PD, according to the MG-RAST analyses. The most abundant Ascomycota in the JC sample was *Nectria haematococca* mp VI 77-13-4, which is a plant pathogen and it is the teleomorph (sexual reproductive stage) of *Fusarium solani*.

**Figure 3:**
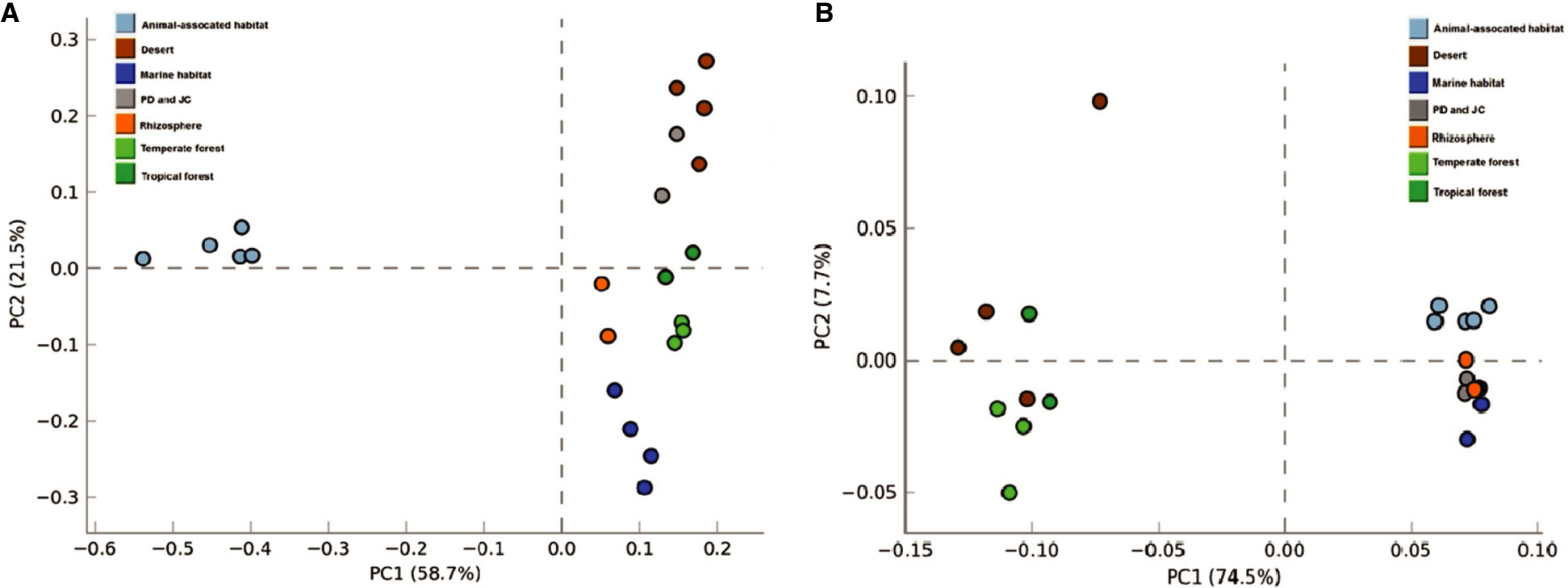
Trends in the PD and JC taxonomy for the class level (A) and for SEED subsystems at level 1 (B) examined using Principal Component Analysis (PCA) through the STAMP software, based on multiple group analysis, applying ANOVA test, Games–Howell post hoc test for confidence interval method and Benjamin–Hochberg FDR for correction.

As the tropical forest and arid soil representatives, the PD and JC samples were compared with public metagenomes. Interestingly, the principal component analysis showed that PD and JC were more similar to each other than to other metagenomes (Fig. 3), and were considered as one group in order to compare with other groups such as rhizosphere and desert.

**Figure 4:**
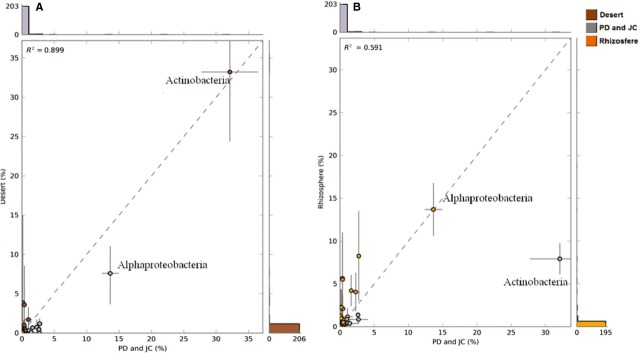
Comparison computed using two group analysis at class taxonomic level for PD and JC versus deserts (A) and PD and JC versus rhizospheres (B). The Welch′s *t*-test, the Welch′s inverted test for confidence interval method and Benjamin–Hochberg FDR for correction were applied. Data related to relative frequency. Significant differences were not observed.

**Figure 5:**
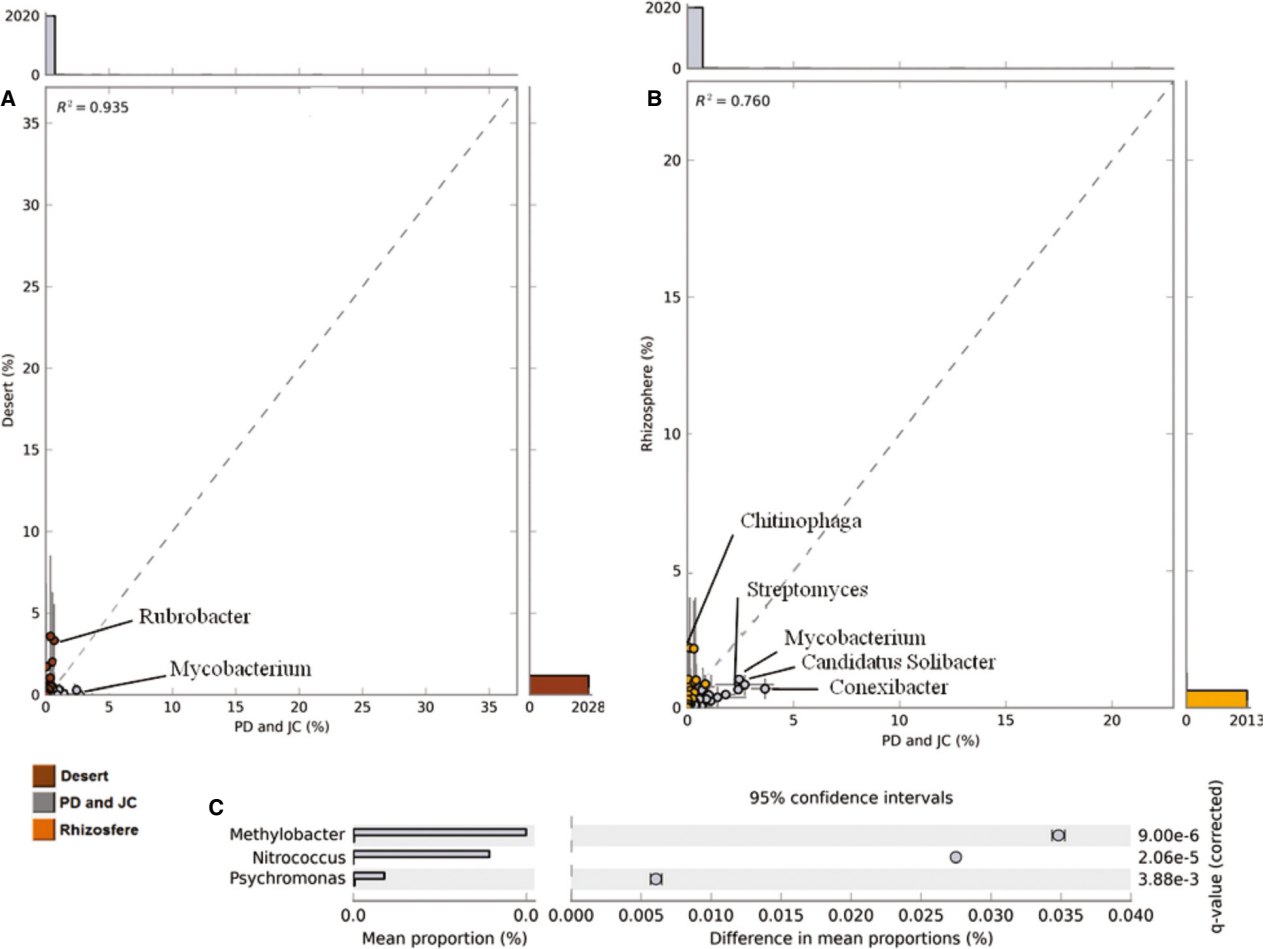
Comparison computed using two groups analysis at genus level for PD and JC versus deserts (A). The significant genera are shown in (B). Genera with difference of relative frequency between PD and JC versus desert (C). The Welch′s *t*-test, the Welch′s inverted test for confidence interval method and Benjamin–Hochberg FDR for correction were applied.

The taxonomic profile indicates that the PD and JC group clustered near desert, with 80.2% and 72.4% of variance being explained by the first two components for class and genus, respectively (Fig. 3A). Although not significant, the PD and JC samples differ from desert soils in the Alphaproteobacteria proportion, which is two times higher in the Brazilian biomes (13.6% and 7.5%) (Fig. 4). In contrast, the desert samples showed a slight increase in Actinobacteria (33.2% and 32.1%) (Fig. 4), especially in relation to the *Rubrobacter* genus (3.3% and 0.6%), and a lower *Mycobacterium* representativeness (0.2% and 2.4%) (Fig. 5). Moreover, the analyses showed that in PD and JC there is an overrepresentation of *Methylobacter, Nitrococcus,* and *Psychromonas* genera (Gammaproteobacteria class), which are statistically relevant (Fig. 5). In deserts, these genera are practically absent.

In comparison with metagenomes from the rhizosphere, PD and JC showed high divergence in the Actinobacteria abundance (32.1% and 7.9%, respectively) (Fig. 4), particularly with *Conexibacter* (3.6% and 0.6%), *Mycobacterium* (2.4% and 1.0%), and *Streptomyces* (2.3% and 0.6%) genera (Fig. 5). In contrast, Rhizosphere presented an elevated proportion of Betaproteobacteria (8.2% and 2.7%), Gammaproteobacteria (4.2% and 1.6%), and Deltaproteobacteria (4% and 2.2%) (Fig. 4). At genus level, a higher abundance of *Chitinophaga* was evident in rhizosphere (2.1% and 0.1%, respectively) (Fig. 5). Despite the divergence observed in the class abundance, it was not statistically significant.

### Comparative functional profiles

**Figure 6:**
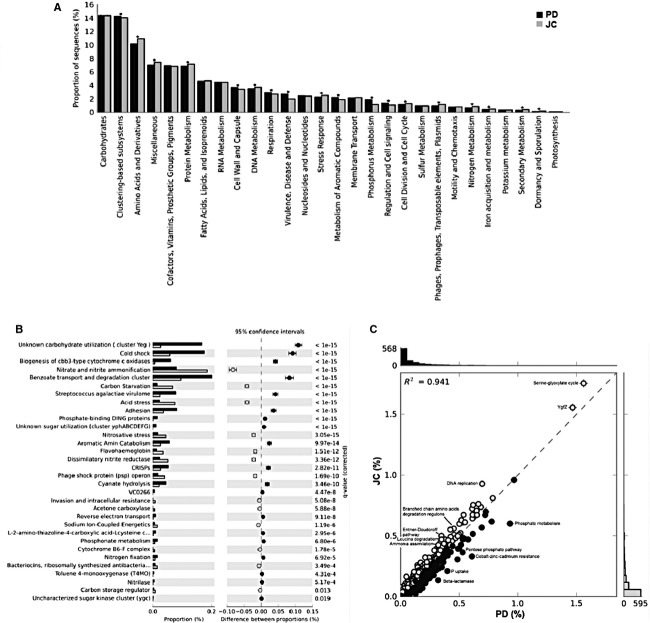
Comparative functional profile of PD (in black) and JC (in gray) samples identified by MG-RAST and statistically analyzed by STAMP. (A) Subsystems at level 1 (*P < 0.05). (B) Subsystems at level 2 (*P* < 0.05, difference between the proportions >1% and a twofold ratio between the proportions). (C) Abundance of subsystems at level 3.

The functional profile obtained using MG-RAST and STAMP did not show discrepant difference in proportion for subsystems at level 1 (considering differences of at least 1% and a twofold ratio between the proportions) (Fig. 6). However, some subsystems of level 1 present a higher representation in PD or JC with a *P* < 0.05 (Fig. 6). In addition, significant differences in proportion were observed at levels 2 and 3. At level 1, the carbohydrates subsystems and clustering-based subsystem, (which groups hypothetical protein families based on conserved colocalization across multiple genomes), were the most abundant in both JC and PD sample. At level 3, the subsystems serine-glyoxylate cycle and YgfZ showed the highest representation. Subsystems such as amino acids and derivatives, DNA metabolism, stress response and nitrogen metabolism are highlighted in JC sample, mainly in relation to genes involved with degradation of amino acids, DNA repair and replication, ammonia assimilation and nitrate and nitrite ammonification. PD sample shows the highest representation of the subsystems virulence, disease and defense, metabolism of aromatic compounds, and phosphorus metabolism, with significant representation of functions related to resistance to antibiotics and toxic compounds, benzoate degradation, and phosphorus uptake (Fig. 6). The analysis of taxonomical distribution among the reads annotated in each subsystem showed that in PD the genera *Rhodopseudomonas, Candidatus solibacter, Candidatus koribacter, Bradyrhizobium, Burkholderia, Nitrobacte,r and Frankia* present the highest contribution for the functional differences observed, while in JC the main genera found were *Mycobacterium, Nocardioides, Rubrobacter, and Burkholderia* (Figure S3). The functional profile obtained using MEGAN was similar to the MG-RAST data (data not shown).

**Figure 7:**
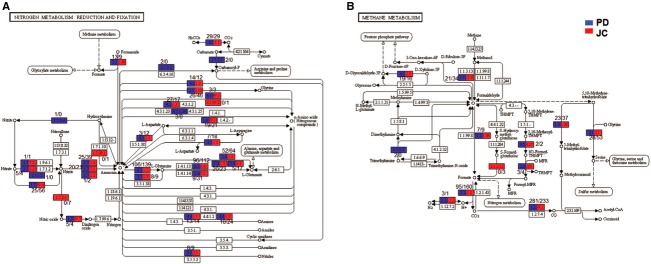
Nitrogen (A) and Methane (B) metabolic pathways performed by KEGG mapper from MG-RAST, with the hit number obtained for each EC number in relation to PD (blue) and JC (red) (adapted by Kanehisa – http://www.genome.jp/kegg).

The biogeochemical cycle analyses evaluated the nitrogen and methane metabolism (Fig. 7), showing that the metagenomes have similar profiles in relation to the enzymes involved in these metabolic pathways, with some exceptions. Concerning to the nitrogen metabolism (Fig. 7A), singular patterns in the proportion of hits were observed in JC. A higher representation of enzymes associated to ammonia production and conversion into amino acids was found. In the methane metabolism cycle (Fig. 7B), PD showed a higher occurrence of the carbon monoxide dehydrogenase (ferredoxin) (EC 1.2.99.2), an enzyme involved in the oxidation of CO to CO_2_, while JC had a higher abundance of catalase (EC 1.11.1.6) and glycine hydroxymethyltransferase (EC 2.1.2.1), which acts in the methylenetetrahydrofolate to serine conversion.

**Figure 8:**
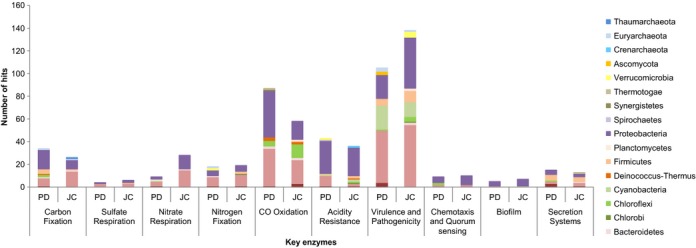
Comparison between PD and JC samples for key enzymes coding genes of distinct metabolic pathways: Carbon Fixation (*abfD, cbbQ, cbbO, cbbX, cbbR*), Sulfate Respiration (*dsrA*), Nitrate Respiration (*nirK, nirS, narG, narH, napA, napB, napC, nosZ*), Nitrogen Fixation (*fixA, fixB, fixC, nifA, nifH*), CO Oxidation (*coxL, coxM, coxS*), Acidity Resistance (*idhD, atpA, aldB, cfA, groEL, pstS, phoBDRSU*), Virulence and Pathogenicity (*katG, aroG, ohr, cpkA, prfA, palFA, sdh1, mpg1, pth11, acr1, ace1, pelA, cut1, pth3, pth8, pkS1-15, tri5, fsr1, magB, mgv1, mps1, pmk1*), Chemotaxis and Quorum sensing (*pilG, pilH, pilJ*), Biofilm (*hnsP*), and Secretion Systems (TIII Type – *escV, pscJ, fliI, flhA*/TIV Type – *virB4, virB11, cpaF*).

The screening of the functional key enzyme (Fig. 8) showed a dominance of Proteobacteria and Actinobacteria hits. Although Acidobacteria was one of the dominant phyla in the PD sample, few hits assigned to this phylum have been identified among the key genes analyzed. The highest number of hits was observed for virulence and pathogenicity, CO oxidation, and acidity. Differences between the samples were identified in the carbon fixation, CO oxidation, and acidity resistance categories, which were predominant in the PD sample, while the virulence, pathogenicity, and nitrate respiration were predominant in the JC sample.

When comparing PD and JC reads with public metagenome considering the functional profiles, the principal component analysis showed that PD and JC were more similar to the rhizosphere metagenome (Fig. 3B), differing from the taxonomic profile which showed PD and JC metagenomes close to desert microbiota (Fig. 3A).

As a group, PD and JC did not present significant categories in comparison with the rhizosphere samples. However, when comparing a rhizosphere sample to PD or JC individually, the rhizosphere metagenomes have more sequences related to the DNA metabolism and iron acquisition and metabolism at level 1, whereas PD presented a higher number of hits related to the carbohydrate and phosphorus metabolism. In the JC sample, the amino acids and derivatives, fatty acids, lipids, and isoprenoids categories are increased (Figure S4).

A significant functional variation was observed at level 1 related to cell wall and capsule; motility and chemotaxis, amino acids and derivatives, nucleosides and nucleotides, and membrane transport, which were all predominant in the PD and JC group (Figure S5) when compared to desert metagenomes.

## Discussion

The soil samples analyzed in this work are classified as sandy soil with very low nutrient content. The data obtained from JC sample are similar to that observed in the majority of the soils from Caatinga biome (recently reviewed by Giongo et al. (2011) and Menezes et al. 2012). In contrast, PD sample differs from other Atlantic Forest soils that show a higher organic matter content and clay proportion with higher water holding capacity (Faoro et al. 2010; Sousa Neto et al. 2011; Vieira et al. 2011). These differences may be associated to the distinct taxonomic and functional profiles discussed below, as soil characteristics such as nutrient availability, moisture, and texture are determinants for microbial diversity and consequent biogeochemical processes (Arias et al. 2005; Chau et al. 2011; Saggar et al. 2013).

The microbial diversity found in PD soil show a distinct profile compared to the Atlantic Forest of Rio de Janeiro and Paraná, also differing from soils of Amazon and Cerrado, where Acidobacteria phylum was dominant, accounting for between 29 and 63% of 16S rRNA sequences obtained (Jesus et al. 2009; Bruce et al. 2010; Faoro et al. 2010; Araujo et al. 2012). Moreover, a high occurrence of Acidobacteria was observed in soils from other subtropical or tropical moist forests (Kim et al. 2012; Nie et al. 2012). It has also been one of the main groups found in arid or semiarid, ranging from 4% to 18% of the sequences of 16S (Chanal et al. 2006; Bachar et al. 2010; Aguirre-Garrido et al. 2012), contrasting with the JC sample, where only 2.5% of the sequences corresponded to the Acidobacteria phylum.

The genome analysis of three Acidobacteria species indicated the presence of cellulose synthesis genes and excreted proteins suggesting potential traits for desiccation resistance (Ward et al. 2009). However, the Acidobacteria physiology is still relatively unknown and data about temperature and ultraviolet resistance are scarce. Furthermore, the discrepancy between our data and those obtained in other Brazilian biomes soils may be related to sand content in PD and JC samples, since the abundance of Acidobacteria was found to be higher in the clay than in the sand or silt fractions (Liles et al. 2010; Russo et al. 2012). Other aspect that is important to consider is the different methodology used for taxonomic analysis. In previous works about Atlantic forest, Amazon and Cerrado, the analysis was based on 16S sequences (Jesus et al. 2009; Bruce et al. 2010; Faoro et al. 2010; Araujo et al. 2012). In this work, our analysis was based on LCA approach of the MG-RAST and MEGAN using the total DNA sequences. The 16S rRNA analysis has been efficient for taxonomic identification, however, expanding the number of markers to include other highly conserved genes has improved the phylogenetic resolution. Methods based on LCA or other parsimonious evolutionary principles are useful to reduce false-positives generated by tools based on homology, increasing the analysis robustness and permitting more precise taxa abundance estimation (Clemente et al. 2010; Guo et al. 2013; Segata et al. 2013).

Our data also showed that Actinobacteria was the dominant phylum in both samples. A high occurrence of Actinobacteria in semiarid soils (20–50%) has been previously reported (Chanal et al. 2006; Bachar et al. 2010; Köberl et al. 2011; Aguirre-Garrido et al. 2012), as observed in JC sample (36.4%). However, its occurrence in forest is generally low (<15%) (He et al. 2006; Lin et al. 2010; Nie et al. 2012), especially in Atlantic forest and Amazon soils (<5%) (Jesus et al. 2009; Bruce et al. 2010; Faoro et al. 2010), differing from PD sample, in which Actinobacteria recorded 27.8% of the hits. Some authors have proposed that in vegetated soil, rhizosphere zone or under plant canopy, the Proteobacteria occurrence is high, while barren soils are characterized by Actinobacteria or Acidobacteria abundance (Bernard et al. 2007; Thomson et al. 2010; Bachar et al. 2012). In sandy soil, Actinobacteria is one of dominant classes found (Russo et al. 2012). Soil water content is a determinant factor for Actinobacteria abundance, which increases in arid soil due to the resistance of several species to drought stress (Connon et al. 2007; Brockett et al. 2012).

Comparative metagenomics analyses have indicated that the substrate (i.e., soil or water) plays a fundamental role in determining the taxonomic and functional profiles of microbial communities. Therefore, soil samples tend to be more similar to each other in relation to taxonomy and the presence of environment-specific genes than samples from other environments (Tringe et al. 2005; Jeffries et al. 2011). In PD, sand and nutrients contents and vegetation seems to be the most important factors for microbial diversity, while in JC, in addition to these same factors, stress conditions (caused by temperature, UV and drought) also affect the microbial diversity. Our samples differ from other soils due to high content of sand and differ from desert biomes due to high occurrence of superior plants. These characteristics may explain the data obtained in our PCA analysis, which showed taxonomic profile of PD and JC similar to desert while the functional profiles were similar to rhizosphere biomes.

Among the genera more represented in PD and JC samples, there are mainly members of Proteobacteria, Actinobacteria, and Acidobacteria phyla. Species of the genera as *Rhodopseudomonas, Bradyrhizobium, Candidatus solibacter, Candidatus koribacter, Nocardioides, Rubrobacter,* and *Geodermatophilus* are described as important in N and C fixation and organic matter degradation, and some of them, as *Rubrobacter xylanophilus* and *Geodermatophilus obscurus* (mainly found in JC sample), are among the most resistant species to gamma and UV radiation (Jothimani et al. 2003; Iwai et al. 2005; Starkenburg et al. 2008; Ward et al. 2009; Ivanova et al. 2010; Chikere et al. 2011; Torres et al. 2011; Yuan et al. 2012). Compared to desert biomes, PD and JC showed a higher occurrence of the genera *Nitrococcus, Psychromonas,* and *Methylobacter*. These genera are described as involved in N and C cycles and stress resistant, including resistance to desiccation and salt stress (Bowman et al. 1993; Koops and Pommerening-Röser 2001; Riley et al. 2008; Ward 2008).

Fungi are also an essential component of terrestrial ecosystems by acting as organic matter decomposers, pathogens and plant-mutualists (Anderson et al. 2003; Hunt et al. 2004; Buée et al. 2009). The higher number of reads found in PD when compared to the JC sample may be explained by the plant diversity found in this biome, since the most represented genera identified are saprophytic or plant pathogens. It has been proposed that fungi are more important for the degradation of complex C source such as cellulose and lignin, while bacteria are more competitive in degrading simple C source. Usually, Basidiomycota is the dominant phylum found in soil samples (Hunt et al. 2004; Buée et al. 2009). This is in contrast to our data that indicated Ascomycota as more frequent, which is mainly attributed to the genus *Aspergillus,* known as saprophytic species and also an opportunistic pathogen (Klich 2002; Horn 2003; Amaike and Keller 2011).

The rapid turnover of organic matter is observed in soils with high temperature and intermediary moisture that are the best conditions for aerobic decomposition, leading to a lower organic matter accumulation and high mineralization rate (Sayer 2006; Fierer et al. 2007), favoring the growth of copiotrophic bacteria as Proteobacteria (Bernard et al. 2007; Thomson et al. 2010). The characteristics of vegetation are an important factor that affects the organic matter decomposition since recalcitrant compounds are more resistant to microbial degradation (Allison and Vitousek 2004; DeAngelis et al. 2011). As representative of the Atlantic forest, PD has a rich diversity of plants, predominantly arboreal, although herbaceous vegetation such as grasses is also found (Freire 1990). Caatinga biome has a lower diversity of plants characterized by deciduous shrubs and xerophilous species (Santos and Santos 2008; Santos et al. 2010, 2012). These characteristics may contribute to the taxonomic and functional profiles found in JC and PD.

The N and P contents in soils are limiting factors, as microorganisms and plants compete for the nutrients. In soils presenting a C:N ratio less than 20:1, the organic matter decomposition occurs quickly, while in soils presenting C:N ratio greater than 20:1, the decomposition is slow (Peng et al. 2002; Rennenberg et al. 2009; Richardson and Simpson 2011). In this condition, the growth of N_2_-fixing bacteria may be favored as observed in the PD sample. Other limiting factor observed in the PD soil is the low phosphorous content that may be related to the highest occurrence of hits annotated in phosphorus metabolism in PD compared to the JC metagenome. These differences may be attributed to genera *Rhodopseudomonas, Candidatus solibacter, Candidatus koribacter, Bradyrhizobium, Burkholderia, Nitrobacter,* and *Fankia*, which presented the highest representation among the hits related to N, C, and P metabolisms compared to JC (Figure S3).

The low nutrient retention in sandy soils suggests that plants play an important role in maintaining the biological diversity due organic matter degradation and/or roots' exudates in rhizosphere that includes compounds that may be used as carbon sources by microorganisms (Bais et al. 2006; Jones et al. 2009). This may explain the highest occurrence of hits associated to the aromatic compounds metabolism and the bacterial defense against toxic compounds, found mainly in PD samples.

In JC soil, the stress caused by high temperatures, UV exposure, and long drought periods seems to be an important trigger factor for microbial diversity. This explains the occurrence of bacteria resistant to stress such as many Actinobacteria genera, as *Mycobacterium, Rubrobacter,* and *Nocardioids*. In fact, the highest frequency of functional categories related to the DNA metabolism, mainly DNA repair and DNA replication, and oxidative and osmotic stress were found JC metagenome. Concerning the N cycle, the nitrate and nitrite ammonification and ammonia assimilation are more represented in the JC metagenome. These are processes possibly related to organic matter decomposition and mineralization (Rennenberg et al. 2009).

Genes related to ammonia oxidation (nitrification-amoA, B and C subunits) were not identified (data not shown) in PD and JC samples, despite the involvement of Bacteria and Archaea species in this N cycle step (Leininger et al. 2006; Di et al. 2009). The low occurrence of genes related to N_2_O production as *NirS* (only 1 hit was found in PD and 1 in JC) suggest a low potential for greenhouse gas production in these soils, as the abundance of this gene was proposed as an indicator of greenhouse gas emission (Morales et al. 2010). Corroborating this hypothesis, a high occurrence of genes involved in nitrite and ammonia assimilation suggests the retention of N and consequently avoiding the loss by denitrification, which is important in an N poor environment.

Additionally, it is interesting to observe that both samples have a good representation of bacteria families described as N_2_-fixer. However, in the PD sample the highest abundance of these bacteria was found, especially when considering the Alphaproteobacteria class, Rhizobiales order. The occurrence of *Bradyrhizobium, Rodopseudomonas,* and *Nitrobacter* genus infers the important role of these microorganisms in N and C fixation and in biodegradation of aromatic compounds (Jothimani et al. 2003; Starkenburg et al. 2008; Torres et al. 2011; Yuan et al. 2012).

Moreover, the CO oxidation genes, especially the CO dehydrogenase, are more represented in the PD sample. The CO oxidation has been used by microorganism as a source of energy and carbon (King and Weber 2007). Soil carbon stocks are affected by addition/decomposition of organic matter. Acidic soils, as observed for PD and JC samples, are generally the most active to remove CO from air (Inman et al. 1971; Bartholomew and Alexander 1979). This explains the occurrence of CO oxidation and acidity-related genes, particularly for the PD sample which has the lowest organic matter content. Furthermore, the higher CO concentration prevents the growth of nitrate-respiring organisms, and the lower oxygen concentration in the soil samples enables some aerobic CO-oxidizers to obtain energy in an anaerobic and nitrate-independent manner (King 2006). An important source for volatile compounds such as CO and CO_2_ is the photodegradation of organic matter (Schade et al. 1999; Brandt et al. 2009), which is a dominant process in semiarid ecosystems during exposure to solar radiation (Austin and Vivanco 2006; Brandt et al. 2007; Day et al. 2007; Gallo et al. 2009). In agreement, JC sample showed the highest occurrence of CO_2_-fixation hits, mainly related to the *Mycobacterium* genus (Figure S3).

Another curious finding is the high occurrence of the gene *abf*D (4-hydroxybutyryl-CoA dehydratase) involved in 3-Hydroxypropionate/4-hydroxybutyrate cycle, a CO_2_ fixation process firstly identified in Archaea species (Berg et al. 2007). It seems to be frequent in Bacteria phyla, while the classical bacteria RuBisCO genes are poorly represented (only 3 hits were found). However, this data should be viewed with caution since the role of these bacterial counterparts still remains unclear (Ettema and Andersson 2008; Ivan et al. 2008).

The subsystem serine-glyoxylate cycle, which is another pathway for C fixation, is well represented in the JC and PD samples and may be associated to organic matter degradation. This is an alternative pathway for mono carbon (C1) assimilation in methylotrophic bacteria as some *Mycobacterium* species. Methanol is very abundant in soil due to degradation of pectin and lignin (Kolb 2009). In addition, YgfZ, a folate-dependent regulatory protein involved in one-carbon metabolism (Teplyakov et al. 2004), is also well represented in both JC and PD. Other indications of this process is the occurrence of genes related to Entner–Doudoroff pathway, as this alternative path for catabolism of glucose to pyruvate was also associated to pectin degradation in some bacteria (Paster and Canale-Parola 1985; Slováková et al. 2002).

In conclusion, even with metagenomics being a powerful tool in the study of microbial biodiversity, much remains to be understood about the biogeochemical processes in soils, requiring a multidisciplinary approach. Although there is a high similarity between the PD and JC samples considering the higher taxonomic and functional levels, significant differences were found in lower hierarchical categories. This was mainly related to the habitat-specific characteristics such as nutrient level, vegetation, and stressful conditions. In both samples, a rapid turnover of organic matter with low greenhouse gas emission was suggested by functional profiles obtained, reinforcing the importance of preserving natural areas. Our data contribute to the understanding of soil microbial diversity in seldom assessed environments up to date.

## Supporting Information

**Figure S1.** Localization of the sampling points: João Câmara city and Parque das Dunas (Natal city), which are situated in Rio Grande do Norte state (Brazil).

**Figure S2.** Analysis of the composition of the PD (black) and JC (gray) communities, showing the Bacteria classes diversity of metagenomic sequences. The data were computed by MEGAN based on a BLASTX using an e-value cutoff of 1e-5. Light gray highlighting on the left side of a node indicates that the up-test of directed homogeneity test showed a significant difference. The thickness of the highlighting is logarithmically proportional to the significance. The size of the bars is scaled logarithmically to represent the number of reads assigned to each taxon.

**Figure S3.** Taxonomical distribution relative to the SEED subsystems that showed differences between PD (black bars) and JC (gray bars). Only genus with the highest representation and significant differences are shown.

**Figure S4.** Comparison of the functional data between: PD versus Holm-Oak rhizosphere (A); PD versus rice rhizosphere (B); JC versus Holm-Oak rhizosphere (C); and JC versus rice rhizosphere; (D) considering subsystem level 1. The relative proportion difference in functional distribution of PD and JC samples was considered significant when *q* < 0.05.

**Figure S5.** Comparison computed using two groups analysis at subsystem level 1 for PD and JC versus deserts. Only significant differences are shown (by Welch´s *t*-test, the Welch´s inverted test for confidence interval method and Benjamin–Hochberg FDR for correction were applied).

**Table S1**. General characteristics of the PD and JC metagenomes.
