# Supplementary figures and images for: Taxonomic and functional profiles of soil samples from Atlantic forest and Caatinga biomes in northeastern Brazil
Source: Microbiologyopen. 2014 Apr 4;3(3):299–315. doi: 10.1002/mbo3.169 (PMC4082704; doi:10.1002/mbo3.169)

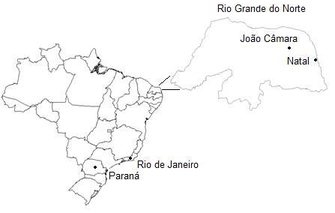

Supplement: Supplementary file 1 — Figure S1. Localization of the sampling points: João Câmara city and Parque das Dunas (Natal city), which are situated in Rio Grande do Norte state (Brazil). [file mbo30003-0299-sd1.jpg]

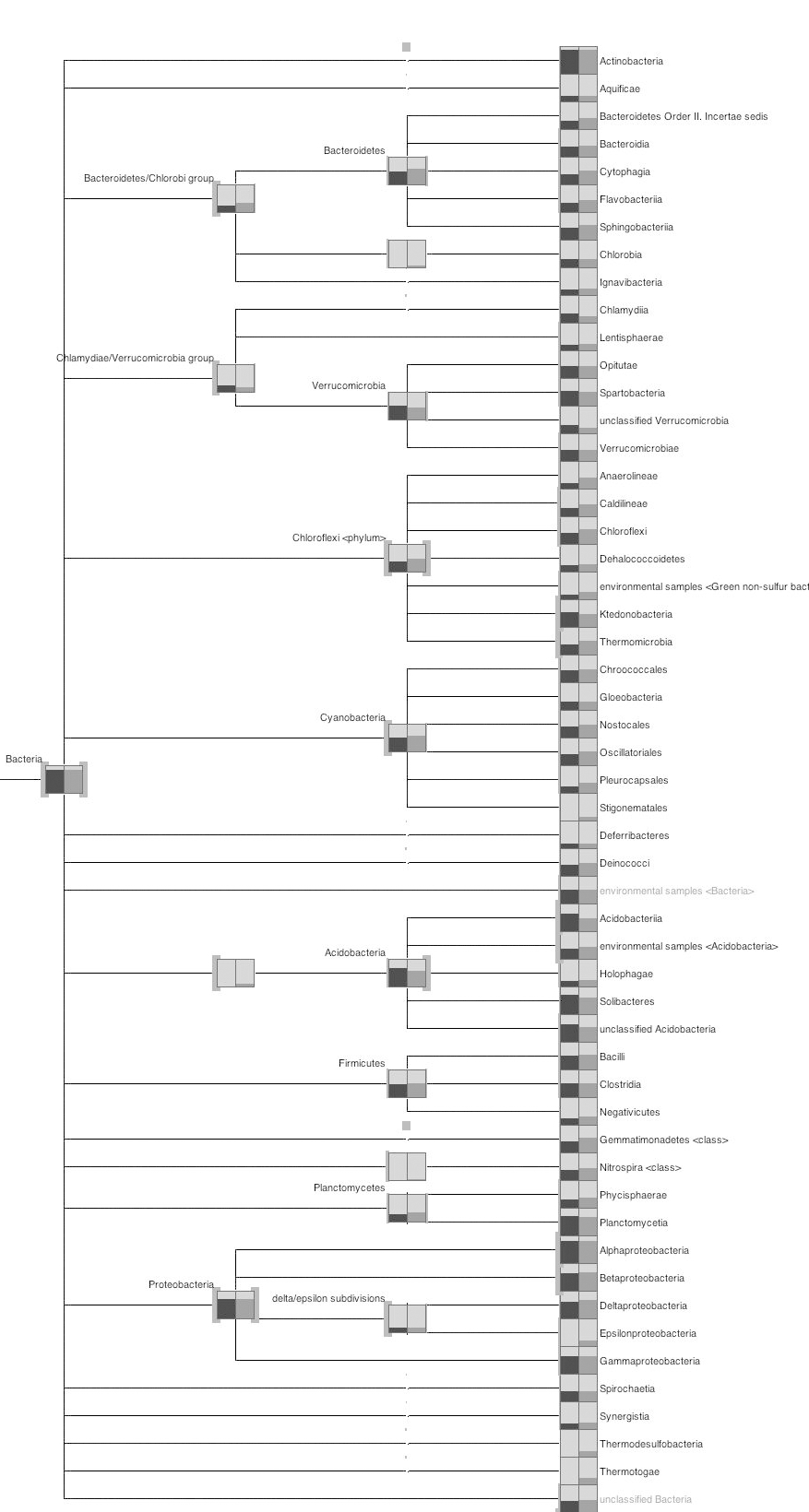

Supplement: Supplementary file 2 — Figure S2. Analysis of the composition of the PD (black) and JC (gray) communities, showing the Bacteria classes diversity of metagenomic sequences. The data were computed by MEGAN based on a BLASTX using an e-value cutoff of 1e-5. Light gray highlighting on the left side of a node indicates that the up-test of directed homogeneity test showed a significant difference. The thickness of the highlighting is logarithmically proportional to the significance. The size of the bars is scaled logarithmically to represent the number of reads assigned to each taxon. [file mbo30003-0299-sd2.jpg]

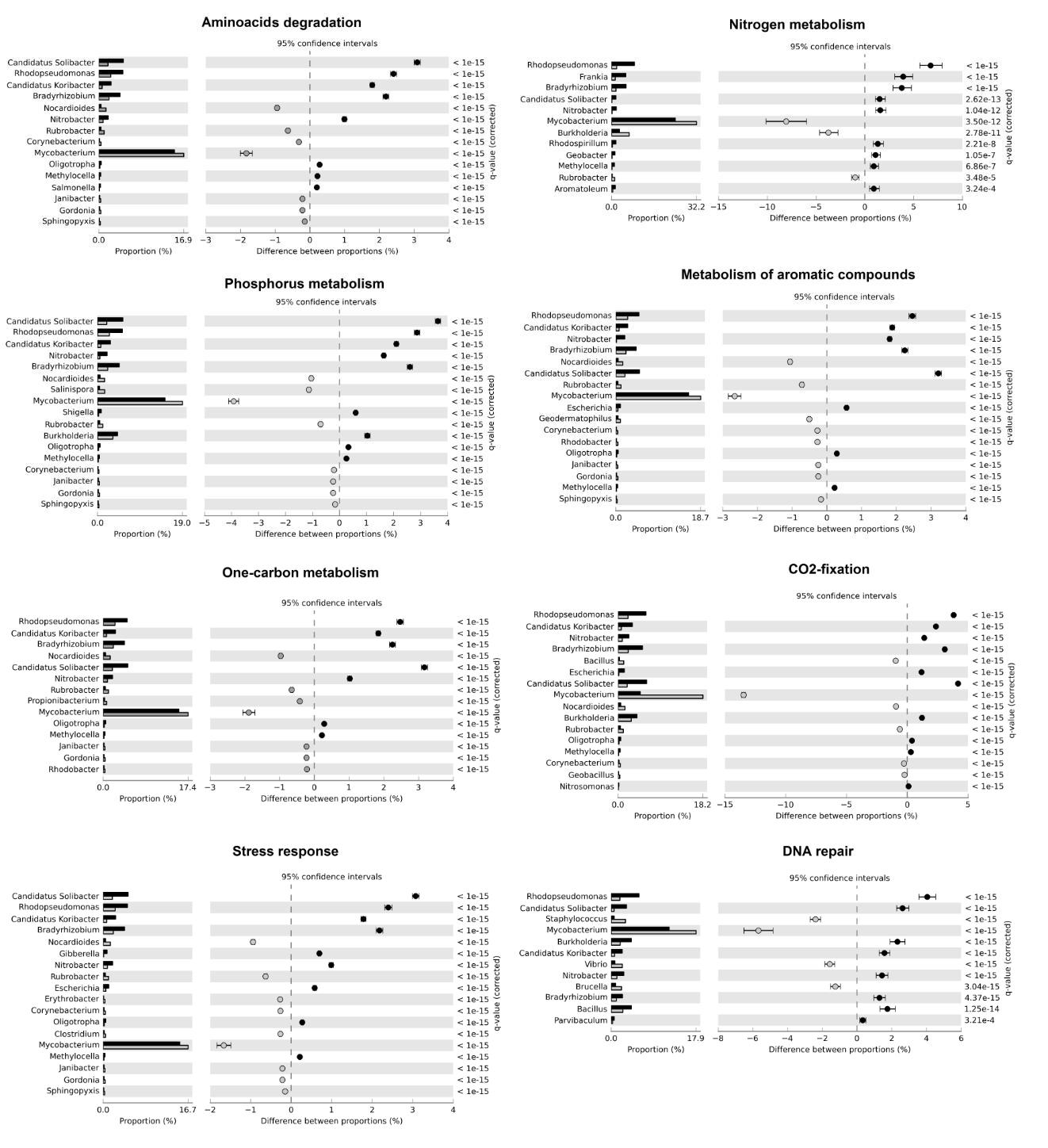

Supplement: Supplementary file 3 — Figure S3. Taxonomical distribution relative to the SEED subsystems that showed differences between PD (black bars) and JC (gray bars). Only genus with the highest representation and significant differences are shown. [file mbo30003-0299-sd3.jpg]

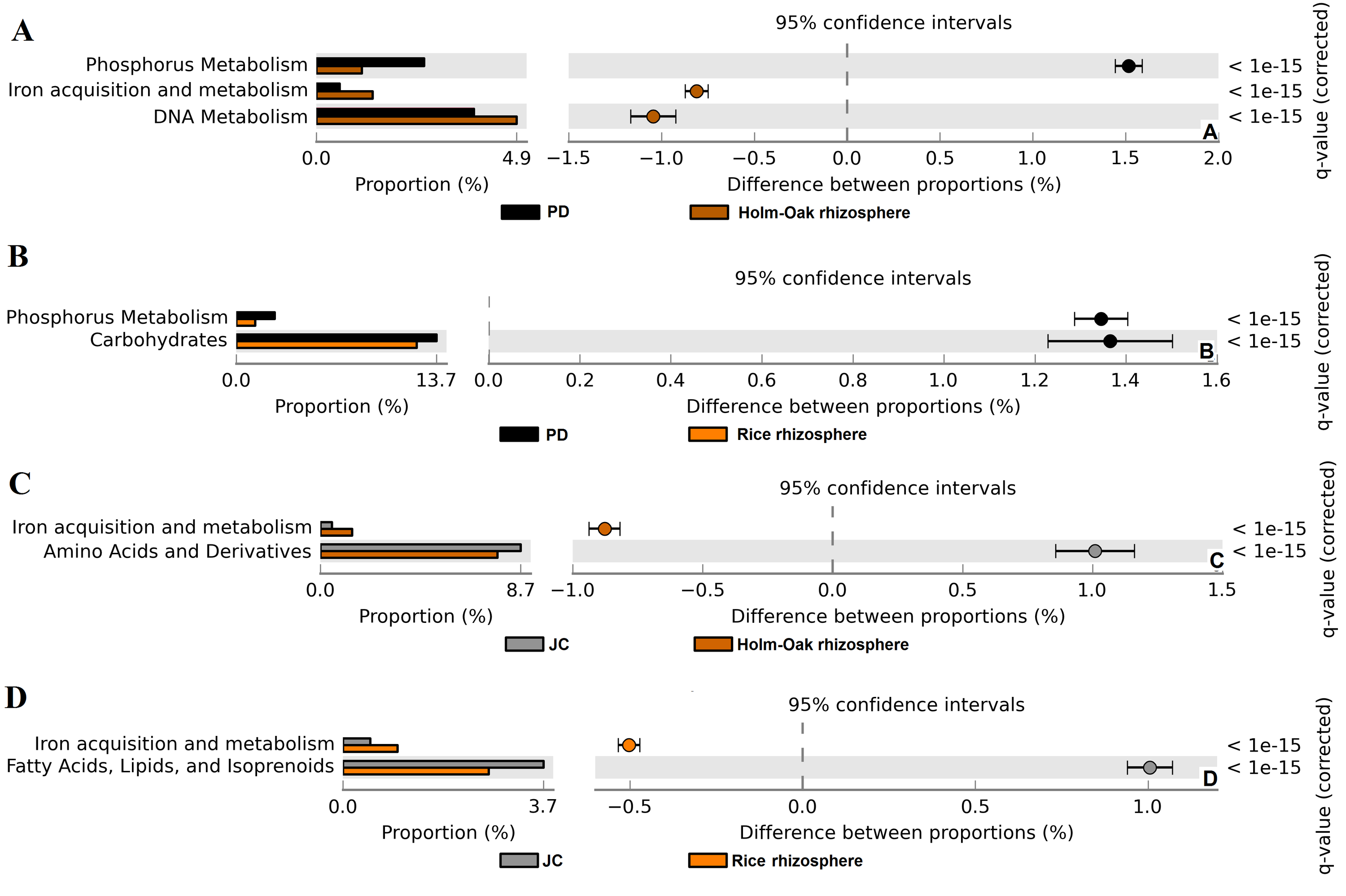

Supplement: Supplementary file 4 — Figure S4. Comparison of the functional data between: PD versus Holm-Oak rhizosphere (A); PD versus rice rhizosphere (B); JC versus Holm-Oak rhizosphere (C); and JC versus rice rhizosphere; (D) considering subsystem level 1. The relative proportion difference in functional distribution of PD and JC samples was considered significant when q < 0.05. [file mbo30003-0299-sd4.jpg]

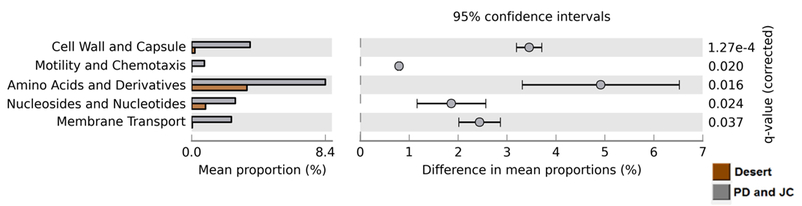

Supplement: Supplementary file 5 — Figure S5. Comparison computed using two groups analysis at subsystem level 1 for PD and JC versus deserts. Only significant differences are shown (by Welch´s t-test, the Welch´s inverted test for confidence interval method and Benjamin–Hochberg FDR for correction were applied). [file mbo30003-0299-sd5.jpg]
