# Supplementary material for: Taxonomic and functional profiles of soil samples from Atlantic forest and Caatinga biomes in northeastern Brazil
Source: Microbiologyopen. 2014 Apr 4;3(3):299–315. doi: 10.1002/mbo3.169 (PMC4082704; doi:10.1002/mbo3.169)
Supplement: Supplementary file 6 — Table S1. General characteristics of the PD and JC metagenomes. [file mbo30003-0299-sd6.docx]

| **Table S1**. General characteristics of the PD and JC metagenomes. | | | | |  |
| --- | --- | --- | --- | --- | --- |
| **MG-RAST** | | |  |  |  |
| **Characteristics** | **PD** | **JC** |  |  |  |
| ID | 4459906.3 | 4459907.3 |  |  |  |
| Number of sequences | 147 278 | 151 274 |  |  |  |
| Average length (bp) | 436 ± 63 | 451 ± 60 |  |  |  |
| Total length (bp) | 64 214 298 | 68 328 253 |  |  |  |
| Mean GC content (%) | 61 ± 8 | 66 ± 7 |  |  |  |
| Predicted proteins coding regions | 155 805 | 164 449 |  |  |  |
| Assigned reads | 84 140 | 99 897 |  |  |  |
| Orphans regions (%) | 46 | 39.3 |  |  |  |

| **MEGAN** | | |
| --- | --- | --- |
| **General characteristics** | **PD** | **JC** |
| Number of sequences | 147 289 | 151 282 |
| Assigned reads | 99 263 | 111 214 |
